# Segmentation of acute pulmonary embolism in computed tomography pulmonary angiography using the deep learning method

**DOI:** 10.5578/tt.20239916

**Published:** 2023-06-13

**Authors:** N. AYDIN, Ç. CİHAN, Ö. ÇELİK, A.F. ASLAN, A. ODABAŞ, F. ALATAŞ, H. YILDIRIM

**Affiliations:** 1 Department of Radiology, Eskişehir Osmangazi University, Eskişehir, Türkiye; 1 Department of Mathematics and Computer Science, Eskişehir Osmangazi University, Eskişehir, Türkiye; 1 Department of Chest Disease, Eskişehir Osmangazi University, Eskişehir, Türkiye

**Keywords:** Artificial intelligence, computed tomography angiography, deep learning, pulmonary embolism, segmentation

## Abstract

**ABSTRACT:**

Segmentation of acute pulmonary embolism in computed tomography pulmonary angiography using the deep learning method

**Introduction:**

Pulmonary embolism is a type of thromboembolism seen in the main pulmonary artery and its branches. This study aimed to diagnose acute pulmonary embolism using the deep learning method in computed tomographic pulmonary angiography (CTPA) and perform the segmentation of pulmonary embolism data.

**Materials and Methods:**

The CTPA images of patients diagnosed with pulmonary embolism who underwent scheduled imaging were retrospectively evaluated. After data collection, the areas that were diagnosed as embolisms in the axial section images were segmented. The dataset was divided into three parts: training, validation, and testing. The results were calculated by selecting 50% as the cut-off value for the intersection over the union.

**Results:**

Images were obtained from 1.550 patients. The mean age of the patients was 64.23 ± 15.45 years. A total of 2.339 axial computed tomography images obtained from the 1.550 patients were used. The PyTorch U-Net was used to train 400 epochs, and the best model, epoch 178, was recorded. In the testing group, the number of true positives was determined as 471, the number of false positives as 35, and 27 cases were not detected. The sensitivity of CTPA segmentation was 0.95, the precision value was 0.93, and the F1 score value was 0.94. The area under the curve value obtained in the receiver operating characteristic analysis was calculated as 0.88.

**Conclusion:**

In this study, the deep learning method was successfully employed for the segmentation of acute pulmonary embolism in CTPA, yielding positive outcomes.

## Abstract (tr)

**ÖZ:**

Derin öğrenme yöntemiyle akut pulmoner embolinin bilgisayarlı tomografik pulmoner anjiografide segmentasyonu

**Giriş:**

Pulmoner emboli, ana pulmoner arterde ve dallarında izlenen tromboemboli çeşididir. Çalışmamızda akut pulmoner emboli tanısını bilgisayarlı tomografik pulmoner anjiyografide (BTPA) derin öğrenme metoduyla koyabilmek, pulmoner embolinin segmentasyonunu yapmak amaçlandı.

**Materyal ve Metod:**

Randevulu çekim yapılan pulmoner emboli tanısı almış hastaların BTPA görüntüleri retrospektif olarak değerlendirildi. Mediasten penceresinde aksiyel kesit görüntüler alındı. Veri koleksiyonu yapıldıktan sonra aksiyel kesit görüntülerde emboli tanısı konulan alanlar segmente edildi. Veri seti eğitim-doğrulama-test olacak şekilde üç parçaya bölündü. Birleşim Üzerinde Kesişim istatistiğinin eşik değeri olarak %50 seçilerek sonuçlar hesaplandı.

**Bulgular:**

Çalışmamıza toplamda 1550 hastadan elde edilen görüntüler dahil edildi. Hastaların yaş ortalamaları 64,23 ± 15,45 yıl idi. Çalışmaya dahil edilen 1550 hastadan elde edilen toplam 2339 adet aksiyel bilgisayarlı tomografi görüntüsü kullanıldı. Pytorch Unet ile 400 epoch eğitildi, en iyi model olan 178 epoch modeli kaydedildi. Test grubunda doğru bulunan, 471; yanlış bulunan, 35; bulunamayan, 27 olarak saptandı. Çalışmamızın sensitivitesi 0,95; precision değeri 0,93; F1 skor değeri 0,94 olarak bulundu. Çalışmaya ait receiver operating characteristics (ROC) analizinde elde edilen AUC değeri 0,88 olarak hesaplandı.

**Sonuç:**

Sonuç olarak çalışmamızda derin öğrenme yöntemi kullanarak akut pulmoner embolinin bilgisayarlı tomografik pulmoner anjiyografide segmentasyonu yapılmış olup başarılı sonuçlar elde edilmiştir.

## Introduction

Pulmonary embolism is a type of thromboembolism seen in the main pulmonary artery and its branches. It is important to make a rapid diagnosis of pulmonary embolism to reduce associated mortality and morbidity ( 1 ). Tests and methods such as D-dimer assay, ventilation-perfusion scintigraphy, lower extremity ultrasonography, and computed tomography pulmonary angiography (CTPA) are used in the diagnosis of pulmonary embolism ( 2-5 ). The CTPA is considered the first-line diagnostic technique in patients with suspected pulmonary embolism ( 6 ). While previously pulmonary angiography was accepted for the diagnosis of pulmonary embolism as the gold standard, computed tomography is accepted as the gold standard in the diagnosis of pulmonary embolism with the development of technology. The CTPA has a higher sensitivity and specificity compared to other examinations ( 7-9 ). However, the radiological interpretation of the CTPA examination is also important. In CTPA, the diagnosis of pulmonary embolism is made by detecting the appearance of filling defects in the arterial lumen and a related increase in diameter, partial filling defects causing the appearance of the polo mint sign and railway track appearance, and peripheral intraluminal filling defects causing acute angulation in the arterial wall ( 6,10 ). The diagnosis of pulmonary embolism involves the examination of the main branches, lobar, segmental, and subsegmental branches throughout the entire lung on CTPA images, which is a timeconsuming process ( 11 ). Therefore, new, and up-to date approaches are needed. Recently, deep learning in artificial intelligence technology has come to the fore in many fields of medicine. Deep learning has components such as lesion detection, classification, segmentation, and quantification, among which segmentation is an important framework used in medical image analysis in structures such as organs and lesions ( 12,13 ). However, only a limited number of studies have reported successful results in the diagnosis of acute pulmonary embolism using deep learning ( 14 ). With the requirement for contemporary approaches and substantiating data, the primary objective of this current study was to utilize the deep learning method for diagnosing acute pulmonary embolism and conducting the segmentation of pulmonary embolism data.

## MATERIALS and METHODS

### Patient Population

After obtaining approval from the ethics committee (15.02.2022, Decision Number: 15), patients presenting to the Eskişehir Osmangazi Medical Faculty Radiology Department between January 1, 2016, and January 1, 2021, were retrospectively screened. Patients aged over 18 years, who underwent scheduled CTPA and were diagnosed with pulmonary embolism, were included in the study.

### Imaging Procedure

The CT scans were performed using 128-section (GE, Revolution EVO) and 64-section (Toshiba, Aquilion) devices with the bolus tracking method and a threshold of 100 Hounsfield Units (HU). Each patient was used to contrast a volume of 60 mL non-ionic with a 100 mL saline chaser at 4.5/5 mL/s. The section thickness of the scans varied between 0.5 mm and 0.625 mm. The images were obtained in the mediastinum window with a width of 300 HU and a height of 50 HU, and the scans were performed at a kV value of 100 and a mA value of 244. The remaining parameters were as follows: detector coverage, 40 mm; rotation time, 0.4 s; pitch, 1.375:1; and speed, 55.00 mm/rot. The sections taken in the mediastinum window were converted to the PNG file format.

### Imaging Analysis

The images were assessed by a radiologist (NA) with seven years of experience and a radiology assistant (ÇC) with three years of experience, reaching a consensus in their evaluations. Complete arterial occlusion with an enlarged artery, the presence of centrally located partial filling defects, and an eccentrically located pulmonary artery exhibiting an acute angle with the vessel wall are indicative of acute pulmonary embolism. Complete arterial occlusion with an enlarged artery, the presence of centrally located partial filling defects, and an eccentrically located pulmonary artery exhibiting an acute angle with the vessel wall are indicative of acute pulmonary embolism. After acute pulmonary embolism was detected in the CTPA images of the patients, axial cross-sectional images were obtained in the mediastinal window. Only images of patients with pulmonary embolism were included in the study. Images with motion artifacts and poor contrast were excluded from the study. Following data collection, the areas that were diagnosed as embolisms in the axial section images were segmented.

The mask images of the labeled regions were generated within the computer science department of our faculty and saved using the same names. Subsequently, the dataset was divided into three distinct groups, namely training, validation, and testing, with proportions of 80%, 10%, and 10% respectively. The mixed-size images were resized to 512 x 512. By applying 50% zoom to the images, the regions to be segmented were enlarged as much as possible to fit the image. The clarity of the regions to be segmented was increased by applying contrastlimited adaptive histogram equalization.

Augmentation was performed on the training and validation groups (both horizontal and vertical), and the amount of data was quadrupled. Epoch training was undertaken using the traditional PyTorch U-Net architecture, which was extended to manage volumetric input. The learning rate of the model was 0.0001. The jump links used between the corresponding encoder and decoder layers allowed for the deep parts of the network to be trained efficiently and facilitated the comparison of the same receiver features with different receiver domains ( 15 ).

The results were calculated by selecting 50% as the cut-off value of the intersection over the union statistic (Jaccard index), which measures the similarity between finite sample sets and is defined as the size of the intersection divided by the size of the union of the sample sets ( 16 ).

### Statistical Analysis

Continuous data were displayed as mean ± standard deviation values, while categorical data were displayed as percentages (%). IBM SPSS Statistics v. 21.0 (IBM Corp. Released 2012. IBM SPSS Statistics for Windows, Version 21.0. Armonk, NY: IBM Corp.) was used to analyze the data. The sensitivity, precision, F1 score, area under the curve (AUC) values, and learning rate were calculated. The F1 score was calculated according to the following formula with true positive, false negative, and false positive values ( 17 ) F1 = 2 * True Positive / (2 * True Positive + False Positive + False Negative)

## RESULTS

Images obtained from 1.550 patients were included in the study. The mean age of the patients was 64.23 ± 15.45 years. A total of 2.339 axial CTPA images obtained from 1.550 patients were used. A total of 5.992 labels were obtained, with 1.879 images and 4.929 labels being used in the training stage, 230 images and 530 labels in the validation stage, and 230 images and 533 labels in the testing stage. By applying augmentation (both horizontal and vertical) to the training and validation sets, the number of data was quadrupled (training set: 7.516 images and 19.716 labels; validation set: 920 images and 2.120 labels) (Table 1. Using PyTorch U-net, 400 epochs were trained, and the best model, epoch 178, was recorded.

**Table 1:** Number of images and labels in the training and validation set

Number of patients	Number of images	Number of labels	Number of images after data augmentation	Number of labels after data augmentation
1.550	1.879	4.929	7.516	19.716
1.550	230	530	920	2.120

**Figure 1:**
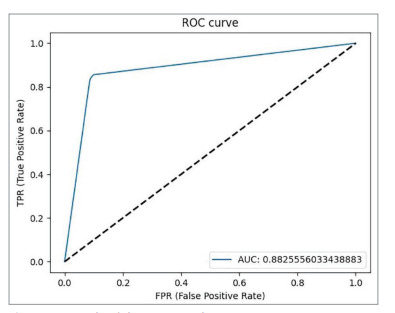
Graph of the ROC analysis (ROC, receiver operating characteristic. AUC: Area under the curve

**Figure 2:**
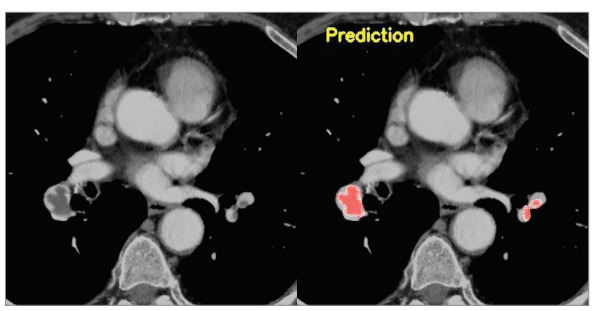
Computed tomographic pulmonary angiography examination revealed filling defects consistent with acute pulmonary embolism in the pulmonary arteries and segment branches of the lower lobes of both lungs in a 63 year-old male patient. Segmentation of the areas consistent with the detected acute pulmonary embolism was performed.

**Figure 3:**
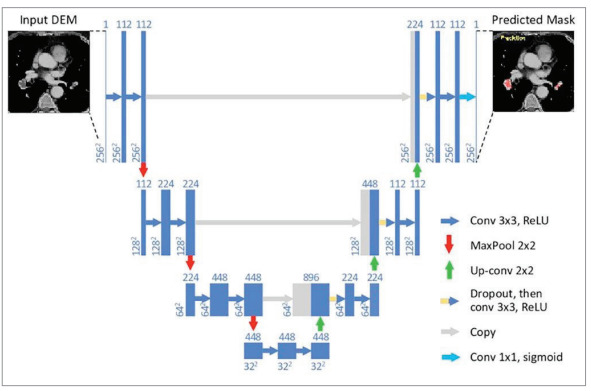
The image of the U-net architecture

In the testing group, the number of true positives was determined as 471 and the number of false positives as 35, while 27 could not be detected. The sensitivity of CTPA segmentation was 0.95, the precision value was 0.93, the F1 score value was 0.94, and the learning rate was 0.0001. In the receiver operating characteristic (ROC) analysis, the area under the curve (AUC) value was calculated as 0.88. The graph of the ROC analysis showing the AUC value is given in Figure 1. The image of the patient whose segmentation was successfully performed is given in Figure 2. The image of the U-net architecture is given in Figure 3.

## DISCUSSION

Artificial intelligence studies are increasing day by day in all fields of science, especially medicine. As a current issue, in our study, we segmented pulmonary embolism using the deep learning method on axial section images of patients with pulmonary artery embolism who underwent CTPA. In the testing group of our study, the sensitivity, precision, F1 score, and AUC values obtained with our artificial intelligence model were measured as 0.95, 0.93, 0.94, and 0.88, respectively, indicating successful results in the diagnosis and segmentation of pulmonary embolism.

Weikert et al. evaluated the performance of an artificial intelligence algorithm called the AI-powered algorithm and detected pulmonary embolisms on CTPA images. The authors used approximately 28.000 CTPA images for validation; and utilized the ResNet architecture in the convolutional neural

network application. The sensitivity value of the AI-powered algorithm in the detection of pulmonary embolism was found to be 92.7%. In contrast, in our study, segmentation was performed instead of detection. Our sensitivity value was 95%, which is slightly better compared to the value reported by Weikert et al. In addition, we did not include the images of patients without pulmonary embolism, unlike the previous study ( 18 ).

In another study using the computer-aided detection algorithm, the sensitivity was calculated separately for the detection of emboli in the main pulmonary artery, lobar, segmental, and subsegmental arteries, and as expected, the sensitivity in the detection of embolism in the main pulmonary artery (87%) was found to be higher compared to the subsegmental artery (61%) ( 19 ). Different from this study, we did not perform separate calculations for embolisms detected in the main pulmonary, lobar, segmental, and subsegmental arteries.

Rucco et al. used the neural hyper network for the diagnosis of pulmonary embolism and obtained data from the images of 1.427 patients. This method successfully diagnosed pulmonary embolism at a rate of 94% ( 20 ), which is quite similar to our study. In another study conducted using the deep learning method, multimodal fusion was used, and both clinical and laboratory data and images of the patients were evaluated ( 21 ). The AUC value of that study was higher than ours. This may be due to the previous authors’ inclusion of clinical and laboratory data in their evaluation.

Huang et al. used the PENet system and attempted to diagnose pulmonary embolism on volumetric CT images with 3D CNN in the infrastructure ( 22 ). The authors found the AUC value to be approximately 0.85, indicating that our method was more successful. In addition, different from our study, Huang et al. performed detection rather than segmentation.

In another study that aimed to detect pulmonary embolism with deep learning, clot burden assessment was also performed, and segmentation was applied ( 23 ). Unlike our study, the authors also calculated the volume of embolism and measured cardiovascular parameters on CT for pulmonary embolism.

Ma et al. trained with RSNA-STR Pulmonary Embolism CT Dataset, and their model was successful in pulmonary embolism detection with a sensitivity of 0.86 and a specificity of 0.85. And they concluded that that model was competitive with the radiologist’s sensitivity and specificity. They proposed multitask learning method that could recognize the presence of pulmonary embolism and its properties such as the position, acute or chronic form, and right-to-left ventricle diameter ratio ( 24 ). In our study, there was only one group that had an acute pulmonary embolism. And our sensitivity result was more successful than their model.

In another study, they compared different deeplearning architectures for pulmonary embolisms. They found CNNs and transfer learning superior to the other methods (25). In our study, we used CNNs algorithm, and we had successful results similar to this study. But we did not compare CNNs with any other algorithm.

We consider that our study is clinically important because the method presented shortens the patient service and evaluation time. In addition, the workload of radiology departments can be reduced using the deep learning-based segmentation model we created, and this will contribute to this field.

One of the limitations of our study is that the segmental branches of the pulmonary artery and the main pulmonary arteries were not considered separately. In addition, the success of the model presented in our study can be increased by including the clinical and laboratory findings of the patients and evaluating cardiovascular parameters that contribute to the course of pulmonary embolism using CT. This will help obtain more successful model alternatives.

## CONCLUSION

In conclusion, in this study, the segmentation of acute pulmonary embolism in CTPA was performed using the deep learning method, and successful results were achieved. We consider that in the future, artificial intelligence-based algorithms will find more place in clinical operations and facilitate the work of clinicians and radiologists.

## Ethical Committee Approval

Ethical approval for this study was obtained from the ethics committee of the Medical University of Eskişehir Osmangazi (Ethics committee decision number: 15, Date: 15 February 2022).

## Conflict of INTEREST

The authors have no conflicts of interest to declare that are relevant to the content of this article.

## AUTHORSHIP CONTRIBUTIONS

Concept/Design: NA, ÖÇ

Analysis/Interpretation: NA, ÇC, AFA, AO

Data acqusition: : HY, FA

Writing: NA

Clinical Revision: NA

Final Approval: NA
